# Rapidly Progressive Spontaneous Spinal Epidural Abscess

**DOI:** 10.1155/2016/7958291

**Published:** 2016-09-05

**Authors:** Abdurrahman Aycan, Ozgür Yusuf Aktas, Feyza Karagoz Guzey, Azmi Tufan, Cihan Isler, Nur Aycan, İsmail Gulsen, Harun Arslan

**Affiliations:** ^1^Neurosurgery Department, Yuzuncu Yıl University Faculty of Medicine, 65040 Van, Turkey; ^2^Neurosurgery Department, Bagcilar Training and Research Hospital, Istanbul, Turkey; ^3^Pediatric Department, Private İstanbul Hospital, Van, Turkey

## Abstract

Spinal epidural abscess (SEA) is a rare disease which is often rapidly progressive. Delayed diagnosis of SEA may lead to serious complications and the clinical findings of SEA are generally nonspecific. Paraspinal abscess should be considered in the presence of local low back tenderness, redness, and pain with fever, particularly in children. In case of delayed diagnosis and treatment, SEA may spread to the epidural space and may cause neurological deficits. Magnetic resonance imaging (MRI) remains the method of choice in the diagnosis of SEA. Treatment of SEA often consists of both medical and surgical therapy including drainage with percutaneous entry, corpectomy, and instrumentation.

## 1. Case

**Figure 1:**
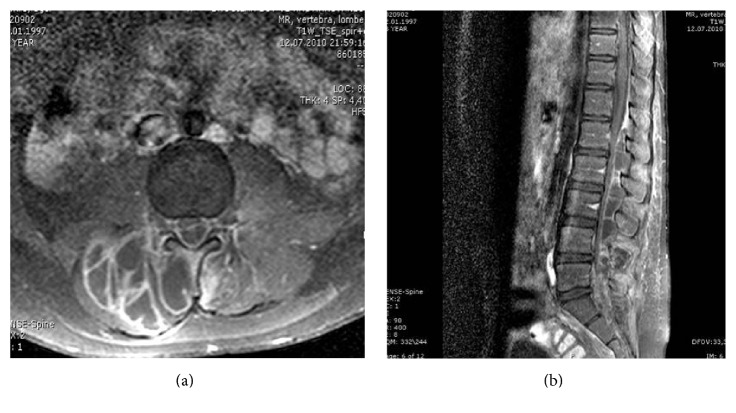
(a) Preoperative axial lumbar MRI. (b) Preoperative sagittal MRI.

A 13-year-old girl presented to our clinic with a 10-day history of low back pain, muscle tenderness, fever, and skin rash. Lumbar MRI showed abscess formation in the paravertebral area at the level of L4-L5 (Figures 1(a) and 1(b)). Initially, the patient refused treatment but after three days, she was readmitted to our clinic due to the increasing complaints including paraparesis in the lower extremities, loss of sensation, and sphincter dysfunction. Systemic examination was normal. Neurological examination revealed a positive Lasègue sign at an angle of 30 degrees, bilateral hypoesthesia at the L3, L4, L5, and S1 dermatomes, paraparesis in the proximal and distal muscle groups of both lower extremities (3/5 and 1/5, resp.), and hypoactive reflex at the lower extremities.

Laboratory parameters were as follows: WBC: 18,500, HB: 5.13, HCI: 34, sedimentation: 80/h, CRP 95/2 h: 115 mg/L, RF: negative,* Salmonella* tests: negative* Brucella* tests: negative and ASO: N. Blood biochemistry and urine analysis were normal. The second thoracic and lumbar MRI scans demonstrated that the paravertebral abscess had spread to the epidural space extending between the levels of S1 and T12.

**Figure 2:**
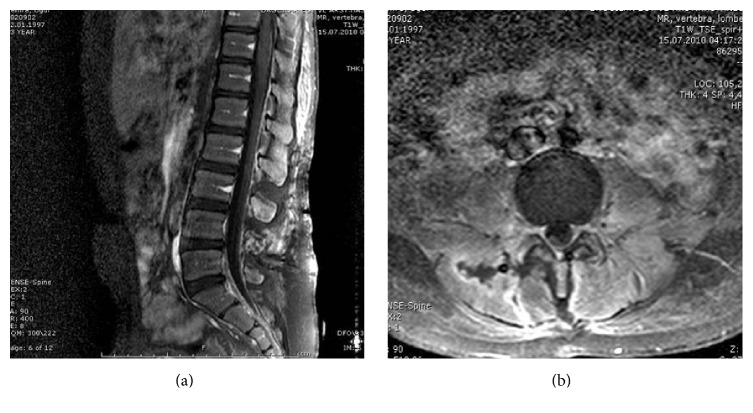
(a) Postoperative sagittal MRI. (b) Postoperative axial MRI with contrast.

The patient was operated on under emergency conditions. Abscess drainage was achieved via the L-1, L-2, L-3, L-4, and L-5 hemilaminectomies and the epidural space was flushed with physiological serum. No bacterial growth was detected in the blood culture. Following the consultation with the Department of Infectious Diseases, an empirical antibiotic therapy including imipenem, aminoglycoside, and Metronidazole was started. Methicillin-resistant* Staphylococcus aureus* (MRSA) was detected in the pus culture of the patient. Depending on the culture-antibiogram results and the consultation with the Department of Infectious Diseases, imipenem was stopped and the antibiotic therapy was restarted with Vancomycin 4 × 500 mg (2 weeks), Penicillin 4 × 1 g, third-generation ceftriaxone 1 g/flk 2 × 1 (14 days), Metronidazole 500 mg flk 2 × 1 (5 days), and Paracetamol suspension (60 days). Rapid clinical improvement was observed within the first days after surgery. In the control lumbar MRI, no abscess was detected in the epidural and paravertebral areas and several scar tissues were seen in the paravertebral area (Figures 2(a) and 2(b)). Pathological examination revealed intensive active chronic nonspecific pus turning to abscess formation in the irregular bones, cartilage, striated muscles, and fat tissue fragments, abundant lipogranuloma formation caused by the macrophages in the fat tissues, and an increase in connective tissues.

**Figure 3:**
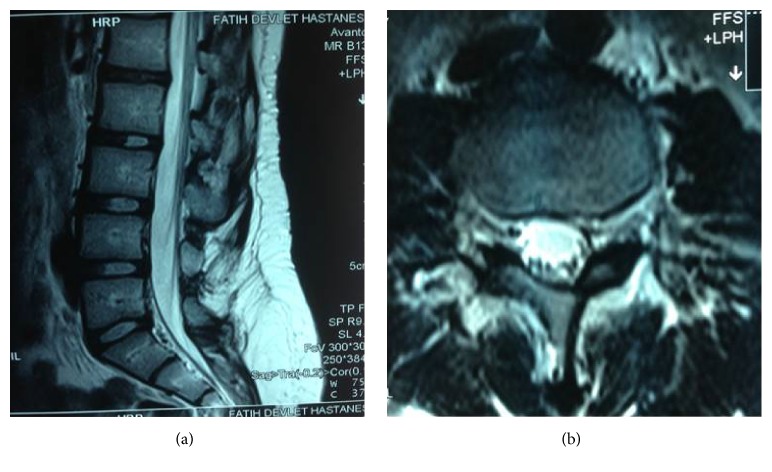
The 3rd year after the operation.

The patient was followed up for 3 years. At annual follow-up visits, no low back pain or sign of infection was detected. No kyphotic increase was observed. The present lumbar MRI images were obtained at follow-up year 3 (Figures 3(a) and 3(b)).

## 2. Discussion

Spontaneous spinal epidural abscess (SEA) is not common in neurosurgical practice. The incidence of SEA is reported to be 1/100,000 individuals, whereas some other studies have reported higher rates [1, 2]. SEA was first reported by Morgagni in 1796 [3, 4] and is usually known as a complication caused by spinal surgery. Epidural abscess may occur with hematogenously spreading infections in another part of the body or through the relation of contamination during surgery, lumbar drainage, and spinal anesthesia or after discography. Most of the SEA patients present with an immunosuppressive disease such as AIDS, diabetes mellitus (DM), chronic renal failure, and tumor. Of these, DM is the most common comorbidity [5]. About 10–20% of the SEA patients present no predisposing factors [6, 7]. Similarly, no predisposing factor was detected in our patient.

SEA is mostly characterized by the fluid collection and inflammatory process on the surrounding dura mater and adipose tissue [8]. The most common strain isolated in SEA patients is* S. aureus*, followed by Streptococci, anaerobic bacteria, and Gram-negative bacilli [9]. Similarly,* S. aureus* was the most common bacterium detected in our patient.

SEA presents three clinical manifestations: acute, subacute, and chronic [10]. The acute symptoms of SEA may be manifested in a few hours or days and are characterized by significant fluid collection. The chronic symptoms of SEA, characterized by inflammatory granular tissue, are relatively slower and may be manifested within weeks or months.

SEA is mostly seen at the thoracic area, followed by lumbar and cervical areas. Common clinical findings of SEA include pain, inflammation, radicular symptoms, spinal cord compression, and the symptoms of cauda equina syndrome [8]. SEA may be mislocalized by the clinical findings and thus the diagnosis of SEA is difficult.

In 1986, three different pathogenic mechanisms were described for paraparesis regarding bacterial infections: (I) with nonspecific polyarthritis, (II) with mass effect of epidural abscess or vertebral collapse related to spondylitis, and (III) with ischemic spinal cord lesion as a result of the abdominal aorta septic thromboembolism [11]. In our patient, depending on their mechanisms of paraparesis, the abscess was considered to be with mass effect.

Magnetic resonance imaging (MRI) remains the method of choice in the diagnosis of SEA [9, 12]. When compared to other methods, MRI provides better outcomes particularly in the early stages of SEA [13, 14].

The primary aims in the treatment of SEA are to identify microorganisms, ensure the drainage of abscess, perform the debridement of granulation tissue, and, if needed, perform spinal stabilization. Treatment of SEA often consists of both medical and surgical therapy including drainage with percutaneous entry, corpectomy, and instrumentation [15]. SEA often is posterior spinal settled. So with hemilaminectomy or laminectomy, it is adequate drainage and debridement. If vertebral osteomyelitis is present, discectomy, corpectomy, or debridement should be performed with stabilization [16].

SEA is a rapidly progressive disease resulting in severe morbidity and mortality in children. Therefore, SEA should be considered in the children presenting with tenderness in the spinal area and pain with or without fever. As in our case, empirical antibiotic therapy is required in SEA patients. In the patients presenting with paravertebral abscess, blood sample should be obtained via abscess drainage and the patient should be strictly monitored during the antibiotic therapy. If neurological deficits or a worsening clinical status appears, radiological imaging should be immediately performed (particularly, MRI) and if neural compression is detected, surgical procedure should be promptly performed. Otherwise, SEA may progress rapidly and may consequently lead to sequela. Paraparesis should be kept in mind in the patients presenting with lumbosacral SEA.
